# Long non-coding RNAs PCAT-1 and FENDRR: diagnostic and prognostic potential in papillary thyroid carcinoma

**DOI:** 10.1186/s12957-025-03863-6

**Published:** 2025-06-04

**Authors:** Aya A. El-Hanafy, Omar A. Ammar, Doaa A. El-Hanafy, Sherine Refat, Amr Sameer, Osama Bahy, Maha O. Hammad

**Affiliations:** 1 Department of Medical Biochemistry & Molecular Biology, Faculty of Medicine, Mansoura University, Mansoura, Egypt; 2 Department of Basic Medical Science, Faculty of Medicine, New Mansoura University, Mansoura, Egypt; 3 Basic Science Department, Faculty of Applied Health Science Technology, Delta University for Science and Technology, Gamasa, Egypt; 4 Department of Biological Sciences, Faculty of Science, New Mansoura University, New Mansoura City, 35742 Egypt; 5 Department of Clinical Pathology, Faculty of Medicine, Mansoura University, Mansoura, Egypt; 6 Department of Pathology, Faculty of Medicine, Mansoura University, Mansoura, Egypt; 7 Endocrine Surgery Unit, General Surgery Department, Mansoura University, Mansoura, Egypt; 8 Department of Surgical Oncology, Faculty of Medicine, Mansoura University, Mansoura, Egypt

**Keywords:** Long non-coding RNA, PCAT-1, FENDRR, Real-time PCR, Papillary thyroid carcinoma

## Abstract

**Background:**

Thyroid cancer is the most prevalent endocrine cancer worldwide. Certain histological subtypes of papillary thyroid carcinoma (PTC) exhibit aggressive behavior, with poor clinical outcomes. Long non-coding RNAs (lncRNAs) have emerged as critical regulators of various biological processes, making them promising candidates for cancer biomarkers.

**Purpose:**

This study aimed to investigate the roles of lncRNAs, specifically prostate cancer-associated transcript 1 (PCAT-1) and Fetal-lethal non-coding developmental regulatory RNA (FENDRR), in the pathogenesis of thyroid carcinoma. Additionally, we evaluated their potential as diagnostic and prognostic markers in PTC.

**Methods:**

Forty fresh thyroid cancer samples and 36 control goiter tissue samples were analyzed for gene expression levels of PCAT-1 and FENDRR via quantitative real-time polymerase chain reaction (PCR), and Associations with clinicopathological characteristics were analyzed. Receiver operating characteristic (ROC) curves were used to evaluate diagnostic accuracy, and the Kaplan-Meier technique was used to analyze survival. A Cox proportional-hazards regression model was used to assess the effects of several risk variables on survival.

**Results:**

Compared with controls, PTC patients predented significantly increased PCAT-1 expression and decreased FENDRR expression, with high diagnostic accuracy according to ROC curve analysis. Patients with lower FENDRR expression had shorter recurrence-free survival, according to Kaplan-Meier analysis. Low FENDRR gene expression, a tumor size greater than 2 cm, invasion into the capsule, and other lobe malignancies remained significant independent prognostic factors for PTC recurrence according to Cox proportional hazards regression analysis.

**Conclusion:**

This study is the first to investigate the expression patterns of PCAT-1 and FENDRR in thyroid cancer. These results indicate that both lncRNAs could be used as diagnostic biomarkers to differentiate PTC from benign thyroid tumors. Furthermore, decreased expression of FENDRR might be used as an independent prognostic factor for predicting PTC recurrence.

## Introduction

Thyroid cancer is the third most common cancer in women [1] and the most common endocrine cancer globally [2]. Advanced thyroid cancer is still a major cause of death even with a generally good prognosis [3]. Identifying biomarkers for early detection and therapy as well as understanding the molecular pathways involved in thyroid cancer are crucial research fields [1].

Over 80% of thyroid malignancies are papillary thyroid carcinomas (PTCs). While most patients respond well to treatment, certain histological variants exhibit aggressive behavior, leading to poor outcomes in up to 30% of cases [4–6]. Distinguishing PTC from benign thyroid lesions, such as nodular goiter or Hashimoto’s thyroiditis, can be challenging due to overlapping histological features [7].

Over the past few decades, substantial advancements have been made in understanding the molecular mechanisms underlying thyroid cancer development, leading to important diagnostic, prognostic, and therapeutic implications. However, this progress has not yet reached an optimal level. Discovering new molecular targets for therapy, as well as molecules for diagnosis and prognosis, holds promise for improving the overall management of this prevalent malignancy [8].

Long noncoding RNAs (lncRNAs) are a family of functional RNA molecules that are longer than 200 nucleotides. These genes are important for controlling gene expression even though they do not encode proteins. Numerous levels of gene regulation, including transcriptional, post-transcriptional, translational, and epigenetic processes, are influenced by these substances [9, 10]. LncRNAs substantially affect signaling pathways related to cell development and cell differentiation while regulating cell proliferation and apoptosis events. The diverse roles of lncRNAs link abnormalities in their expression to many human diseases including cancer [3, 9].

The essential role that lncRNAs play in controlling various biological processes makes them highly suitable for use as cancer biomarkers. Studies have shown that prostate cancer-associated transcript 1 (PCAT-1) functions as an oncogenic factor initially described in prostate cancer patients [11]. Research has revealed that this RNA is involved in multiple human cancer types ranging from hematological cancers to solid tumors. In animal studies, the inhibition of PCAT-1 expression has proven effective as a tool to diminish tumor development across multiple cancer disorders including colorectal cancer, lung cancer, hepatocellular carcinoma, and squamous cell carcinoma [10].

Another recently identified lncRNA is fetal-lethal non-coding developmental regulatory RNA (FENDRR), sometimes known as FOXF1-AS1. FENDRR is located on the long arm of chromosome 3, and consists of four exons and 3,099 nucleotides. This lncRNA displays abnormal expression patterns between different cancer types. Recent research has shown that FENDRR functions as a vital lncRNA that controls cancer initiation and progression [12].

Despite increasing interest in the importance of lncRNAs in cancer research, the precise functions of PCAT-1 and FENDRR in thyroid cancer have not yet been investigated. The goal of this research was to explore the potential roles of both PCAT-1 and FENDRR lncRNAs in thyroid cancer by comparing their gene expression levels between PTC patients and control individuals. This study also aimed to evaluate PCAT-1 and FENDR as prognostic indicators for predicting the recurrence of thyroid cancer and to test their ability to discriminate between PTCs and benign thyroid tumors.

## Subjects and methods

### Sample size

A G*Power 3.1.9.7 (2020) sample size calculator was used to establish the study’s sample size. The calculated sample size of the study was 36 contributors for each group at the 5% significance level and 80% power of the study. According to a pilot study involving 10 patients and 10 controls, elevated RQ_PCAT-1 expression was estimated in 80% of the patient group and 50% of the control group.

### Patients

A total of forty patients who underwent thyroidectomies at the Mansoura Oncology Center, Mansoura University Hospital (Mansoura, Egypt) between March 2023 and April 2024 following a pathological diagnosis of PTC were included in the study. Additionally, thirty-six patients with goiter formed the control research group. The ages of all the participants ranged between 20 and 80 years. The identification of all PTC patients relied on preoperative FNAC and ultrasound testing until postoperative histopathological tests confirmed their diagnosis. Patients with other neoplastic lesions and those who received thyroid-related medicines before surgery were excluded from the research study. Before specimen collection, each participant enrolled in the present study provided informed written consent. This study was approved by the Institutional Research Board (IRB) Committee of the Faculty of Medicine, Mansoura University (Code number: R.23.07.2279).

Clinical data, which included patients’ age at diagnosis and sex, were obtained from medical records. The laboratory data included thyroid function profiles together with blood analysis of erythrocytes, leukocytes, platelets, and lymphocytes. Clinicopathological data for PTC patients, including pathological type, tumor size, tumor stage, multifocality, node metastasis, lymph vascular invasion, and extra-thyroid extension, as well as any additional data regarding recurrence were also obtained from hospital patients᾽ records. According to the 2017 criteria of the American Joint Committee on Cancer (AJCC), tumor staging was carried out [13]. Hospital records were also searched for radiological findings from ultrasonography, including calcifications, mass boundaries, and echogenicity.

### Sample collection

Fresh tissue samples (50–100 mg) were collected from the central portion of thyroid tumor tissue immediately after thyroidectomy, avoiding areas of hemorrhage, necrosis, or fibrosis. The tissue samples were immediately preserved in an appropriate volume of RNA later (Qiagen, Germany, Cat. No. 76104) with approximately10 µL of the reagent per 1 mg of tissue to guarantee RNA integrity. After being kept at 4 °C for at least 24 h, the samples were moved to -80 °C for qRT-PCR analysis. To confirm the diagnosis and evaluate pathological parameters, a different subset of the tissue samples was sent to the pathology department for histopathological analysis. Only thyroid tissue samples were collected during surgery for this study. Malignant tissues were obtained from patients diagnosed with thyroid carcinoma, and benign tissues from patients with nodular goiter were used as controls. Lymph node tissues, even when surgically dissected, were not collected nor processed for molecular or expression analyses.

### mRNA quantification by quantitative real-time PCR (qRT-PCR)

The QIAzol reagent (Qiagen, Germany) was used to extract total RNA from thyroid tissue samples according to the manufacturer’s instructions. The concentration and purity of the RNA were assessed via a NanoDrop 2000 spectrophotometer (Thermo Scientific, USA). For additional analysis, only samples with A260/A280 ratios between 1.80 and 2.00 were used. The extracted RNA was reverse transcribed at -80 °C.

A COSMO cDNA Synthesis Kit (WF-10205002, Willowfort, UK) was used to reverse transcribe 500 ng of total RNA according to the manufacturer’s instructions for cDNA synthesis. The reaction was performed via a thermal cycler (Applied Biosystems, Waltham, Massachusetts, USA) with the following program: 5 min at 25 °C, 15 min at 45 °C, and 5 min at 85 °C, in a 20 µL reaction mixture. The synthesized cDNA samples were stored at − 20 °C.

Using the HERA SYBR Green PCR Master Mix (WF10304001, Willowfort, UK), quantitative real-time PCR was carried out. Each 20 µL PCR reaction included 10 µL of HERA SYBR Green PCR Master Mix, 6 µL of RNase-free water, 1 µL of forward primer (1 µM), 1 µL of reverse primer (1 µM), and 2 µL of cDNA template. An applied Biosystems 7500 Real-Time PCR System (Life Technologies, USA) was used for amplification and fluorescence detection with the following protocol: initial denaturation at 95 °C for 2 min, followed by 40 cycles of 95 °C for 10 s and 60 °C for 30 s.

The mRNA levels of PCAT-1 and FENDRR were normalized to the expression of the housekeeping gene β-actin. The primer sequences used for qRT-PCR were as follows: PCAT-1:

forward: 5′-GCTGGCATTGGTCAACATAAC-3′, reverse: 5′-GTGAATATGGCGGATGAGGAA-3′; FENDRR: forward: 5′-AGCCTACTCGTCAAAAGCCC-3′, reverse: 5′-GCCTAGATCCGAAGGCTGTC-3′, and β-actin: forward: 5′-GTGGCCGAGGACTTTGATTG-3′, reverse: 5′-GTGGGGTGGCTTTTAGGATG-3′.

The primer sets were designed via Primer 3 software (v.4.1.0) [http://primer3.ut.ee] and synthesized by Vivantis Technologies (Malaysia). Primer specificity was verified using the Primer-BLAST tool [https://www.ncbi.nlm.nih.gov/tools/primer-blast/] and confirmed through melting curve analysis.

### Interpretation of the results

The comparative threshold approach (ΔΔCt) was used to evaluate relative gene expression [14]. The relative quantification (RQ) of the target mRNA, normalized to that of the control samples, was the outcome of calculating the relative expression using the cycle threshold (CT) values. The following formula was used to determine the fold change: RQ = 2^−ΔΔCt^.

### Statistical analysis

Version 26 of the Statistical Package for Social Sciences (SPSS) for Windows was used to analyze the data. The Kolmogorov-Smirnov one-sample test was used to determine whether the data were normal. Percentages and numbers were used to describe the qualitative data. The chi-square test was used to examine relationships between categorical variables, and when the expected cell count was less than five, Fisher’s exact test and Monte Carlo tests were used. The continuous variables are displayed as the median (minimum-maximum) for non-normal data and mean ± standard deviation (SD) for normally distributed data. The independent t-test (parametric) or the Mann-Whitney test (nonparametric) was used to compare the two groups. Using ROC curve analysis, sensitivity and specificity at various cutoff points were assessed. The Kaplan-Meier test was used for survival analysis, and the log-rank test was used to determine the statistical significance of the differences between curves. The impact of different risk factors on survival was examined using the Cox proportional hazards regression model. All the statistical tests were conducted with a significance threshold of 5% (p-value). Higher significance was indicated by smaller p-values, and results were deemed significant when *p* ≤ 0.05.

## Results

### Clinical, demographic, and laboratory data of all study groups

A summary of each participant’s routine clinical, demographic, and laboratory data is provided in Table 1. There were 40 PTC patients and 36 goiter patients (control group) included in the study. The mean age of the PTC group was 44.82 ± 16.07 years, with 28 patients (70.0%) being female. The mean age of the goiter group was 39.50 ± 9.51 years, with 33 patients (91.7%) being female. Compared with the goiter group, the PTC group exhibited significantly higher serum total T3 levels (*p* = 0.008), white blood cell (WBC) counts (*p* ≤ 0.001), and platelet counts (*p* = 0.005). Also, PTC patients exhibited significantly lower red blood cell (RBC) counts (*p* ≤ 0.001), hemoglobin levels (*p* ≤ 0.001), and lymphocyte percentages (*p* = 0.002).

**Table 1:** Clinic-demographic characteristics and laboratory investigations of the studied groups

Parameters	Patients group (no = 40)	Control group (no = 36)	Test of significance	*P*-value
Clinic-demographic data				
Age (Years)	44.82 ± 16.07	39.50 ± 9.51	t = 1.73	0.087
Age (years) < 55 ≥ 55	29 (72.5%) 11 (27.5%)	33 (91.7%) 3 (8.3%)	χ^2^ = 0.68	0.41
Sex Male Female	12 (30.0%) 28 (70.0%)	3 (8.3%) 33 (91.7%)	χ^2^ = 1.86	0.172
BMI	31.01 ± 6.33	32.72 ± 6.45	t = 1.11	0.270
Laboratory investigations				
Serum total T3 (*nmol/L*)	1.51 (0.98–4.95)	1.40 (0.46–2.99)	Z = 2.64	0.008*
Serum total T4 (μg/dL)	8.20 (1.97–13.60)	7.72 (1.00-12.83)	Z = 1.18	0.358
Serum TSH level (mIU/L)	1.83 (0-70.30)	1.30 (0.04–11.56)	Z = 0.247	0.805
WBC count (×10^3^ cells/µL)	9.02 ± 3.02	6.63 ± 1.62	t = 4.21	≤ 0.001*
RBC count (×10^6^ cells/µL)	4.21 ± 0.84	4.81 ± 0.44	t = 3.81	≤ 0.001*
Hemoglobin (g/dl)	11.09 ± 1.81	12.41 ± 1.41	t = 3.51	≤ 0.001*
Platelet count (×10^3^ cells/µL)	259.80 ± 88.06	213.27 ± 39.95	t = 2.91	0.005*
Lymphocytic percent (%)	27.27 ± 13.85	36.11 ± 8.39	t = 3.16	0.002*
lncRNA expression levels (fold change)				
PCAT1	3.12 (0.40–7.29)	1.49 (0.23–3.02)	Z = 5.18	≤ 0.001*
FENDRR	0.31 (0.01–1.24)	1.16 (0.35–3.19)	Z = 5.46	≤ 0.001*

t: Independent samples Z: Mann-Whitney U test (data are shown as median and IQR), t-test (data are shown as mean ± SD), and chi-square test (data are shown as count and percent). * A significant difference is shown by *P* ≤ 0.05. Body mass index (BMI); white blood cells (WBCs); red blood cells (RBCs); hemoglobin (Hg); long non-coding RNA (lncRNA); prostate cancer-associated transcript 1 (PCAT1); and fetal-lethal non-coding developmental regulatory RNA (FENDRR)

### Characteristics of patients with PTC

The radiological (ultrasound) and pathological features of the PTC patients are detailed in Table 2. Among the 40 patients included in the study retrosternal extension was observed in 3 patients (7.5%). Ultrasound findings show hypoechoic lesions were present in 32 patients (80%), ill-defined borders were noted in 25 patients (62.5%), and calcifications were detected in 28 patients (70%).

According to surgical techniques, 26 patients (65.0%) underwent complete thyroidectomy with lymph node (LN) dissection, 10 patients (25.0%) underwent total thyroidectomy alone, and 4 patients (10%) underwent hemithyroidectomy. Pathological findings show classic PTC was identified in 35 patients (87.5%), follicular variant PTC was found in 4 patients (10%), and cribriform variant PTC was observed in 1 patient (2.5%). Tumor location detection shows the lesion was detected in one lobe in 28 patients (70.0%), two lobes were involved in 10 patients (25%), and the isthmus was involved in 2 patients (5%). Tumor size measurements show lesions ≤ 2 cm were found in 18 patients (45.0%), capsular invasion was present in 17 patients (42.5%), and lymphovascular invasion was detected in 23 patients (57.5%). TNM staging (AJCC 8th edition) shows stage I: 28 patients (70.0%), stage II: 7 patients (17.5%), and stage III: 5 patients (12.5%).

A surgically excised and pathologically confirmed local lesion or regional/distant metastasis seen by imaging at least six months following first therapy was considered a disease recurrence. Recurrence was observed in 17 patients (42.5%) during the 5-year (60-month) follow-up period.

**Table 2:** Radiological and pathological features of patients with PTC

Patients group (no = 40)	
Radiological features	
Retrosternal extension	
Present	3 (7.5%)
Absent	37 (92.5%)
Mass borders	
Ill-defined	25 (62.5%)
Well-defined	15 (37.5%)
Mass	
Hypoechoic	32 (80.0%)
Isoechoic	8 (80.0%)
Calcification	
Present	28 (70.0%)
Absent	12 (30.0%)
Pathological features	
Operation	
Thyroidectomy + LN dissection	26 (65.0%)
Total thyroidectomy	10 (25.0%)
Hemi thyroidectomy	4 (10.0%)
Site	
One lobe	28 (70.0%)
Two lobes	10 (25.0%)
Isthmus	2 (5.0%)
Size (cm)	
≤ 2	18 (45.0%)
> 2	22 (55.0%)
The other lobe	
Free	19 (47.5%)
MNG	10 (25.0%)
Malignancy	10 (25.0%)
Lymphocytic thyroiditis	1 (2.5%)
Capsule	
Free	23 (57.5%)
Invaded	17 (42.5%)
Lymph vascular invasion	
Present	23 (57.5%)
Absent	17 (42.5%)
Multifocality	
Unifocal	20 (50.0%)
Multifocal	20 (50.0%)
Extra thyroid extension	
Present	7 (17.5%)
Absent	33 (82.5%)
Type	
Classic	35 (87.5%)
Cribriform	1 (2.5%)
Follicular	4 (10.0%)
pT	
T1	12 (30.0%)
T2	23 (57.5%)
T3	5 (12.5%)
pN	
N0	19 (47.5%)
N1	21 (52.5%)
pM	
M0	36 (90.0%)
M1	4 (10.0%)
TNM Stage	
I	28 (70.0%)
II	7 (17.5%)
III	5 (12.5%)
Recurrence	
Present	17 (42.5%)
Absent	23 (57.5%)

Abbreviations: MNG, multinodular goiter; N, nodal metastasis; M, distant metastasis; T, tumor size

### Analysis of PCAT-1 and FENDRR gene expressions in thyroid tissue specimens

PCAT-1 and FENDRR expression levels were assessed in tissue samples from both PTC and goiter. PCAT-1 expression was significantly higher in PTC (median: 3.12; IQR: 0.40–7.29) compared to goiter (median: 1.49; IQR: 0.23–3.02; *p* ≤ 0.001). In comparison to goiter (median: 1.16; IQR: 0.35–3.19; *p* < 0.001), FENDRR expression was significantly lower in the PTC group (median: 0.31; IQR: 0.01–1.24). (Table 1).

### ROC curve analysis

**Fig. 1:**
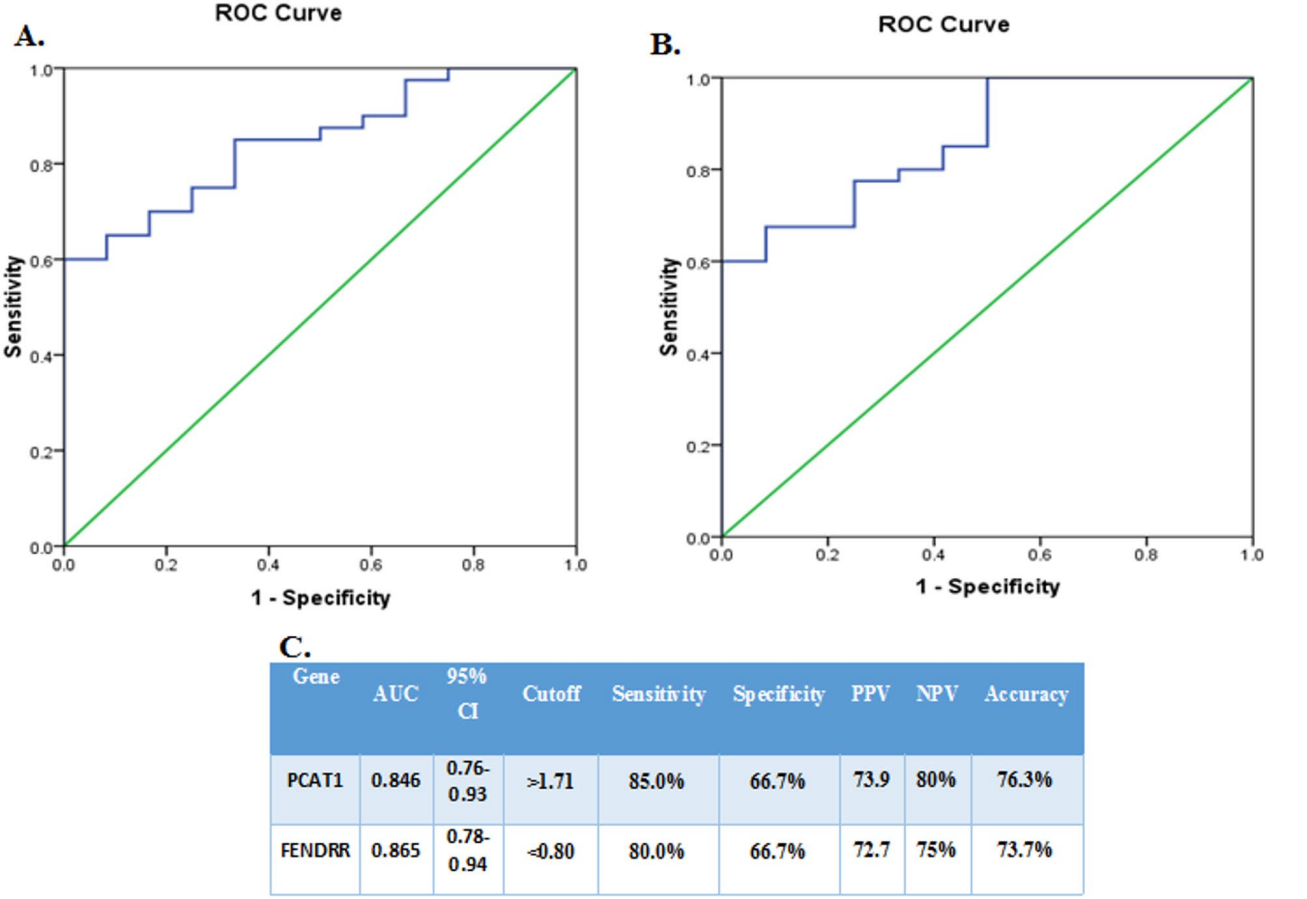
Receiver operating characteristic (ROC) curves of PCAT1 and FENDRR lncRNA levels for the discrimination of PTC and goiter. **A**) ROC curve of PCAT1 with AUC of 0.846. **B**) ROC curve of FENDRR of AUC of 0.865. **C**) Validity of PCAT1 and FENDRR gene expressions to discriminate PTC and goiter. *Abbreviations: Prostate cancer-associated transcript 1 (PCAT1)*,* fetal-lethal non-coding developmental regulatory RNA (FENDRR)*,* the area under the curve (AUC)*,* confidence interval (CI)*,* positive predictive value (PPV)*,* and negative predictive value (NPV)*

The diagnostic accuracy of PCAT-1 and FENDRR expression levels in distinguishing PTC from goiter was assessed using ROC curve analysis. PCAT-1 expression had an AUC of 0.846 (95% CI: 0.76–0.93), a cutoff value of ≥ 1.71, a specificity of 66.7%, and sensitivity of 85%. FENDRR expression had an AUC of 0.865 (95% CI: 0.78–0.94), cutoff value of ≤ 0.80, specificity of 66.7%, and sensitivity of 80% (Fig. 1).

### Association between PCAT-1 and FENDRR gene expressions and patients’ characteristics

The associations between PCAT-1 and FENDRR expression levels and clinicopathological characteristics were analyzed. According to PCAT-1 expression, patients were divided into groups with high (≥ 1.71) and low (< 1.71) expression. High PCAT-1 expression was observed in 85% (34/40) of PTC specimens. High PCAT-1 expression was significantly associated with higher BMI (*p* = 0.021), higher WBC count (*p* = 0.035), classic PTC type (*p* = 0.019), and multifocality (*p* = 0.02) (Table 3).

**Table 3:** Association between the PCAT1 gene expression and patients’ characteristics

Parameters	PCAT1 lncRNA expression (fold change)		Test of significance
	**Low (< 1.71) (n = 6)**	**High (≥ 1.71) (n = 34)**	
Clinic-demographic data			
Age (Years)			
< 55	6 (20.7%)	23 (79.3%)	χ^2^ = 2.67
≥ 55	0 (0%)	11 (100%)	*P* = 0.102
Sex			
Male	2 (16.7%)	10 (83.3%)	FET
Female	4 (14.3%)	24 (85.7%)	*P* = 1.0
BMI	25.73 ± 3.39	32.18 ± 6.26	t = 2.42 *P* = 0.021*
Laboratory investigation			
Serum total T3 (*nmol/L*)	1.44 (0.90–1.56)	1.40 (0.46–2.99)	Z = 0.307 *P* = 0.759
Serum total T4 (μg/dL)	7.10 (6.70–10.50)	8.30 (1.97–13.60)	Z = 0.528 *P* = 0.598
Serum TSH level (mIU/L)	2.00 (0.52–2.60)	1.70 (0.0- 70.30)	Z = 0.195 *P* = 0.846
WBC count (×10^3^ cells/µL)	6.64 ± 2.18	9.44 ± 2.97	t = 2.18 *P* = 0.035*
RBC count (×10^6^ cells/µL)	4.53 ± 0.28	4.15 ± 0.89	t = 1.02 *P* = 0.313
Hemoglobin (g/dl)	12.15 ± 1.66	10.91 ± 1.79	t = 1.57 *P* = 0.123
Platelet count (×10^3^ cells/µL)	315.17 ± 138.47	250.03 ± 74.86	t = 1.71 *P* = 0.095
Lymphocytic percent (%)	34.28 ± 11.24	25.71 ± 14.07	t = 1.39 *P* = 0.175
Radiological features			
Retrosternal extension			FET *P* = 1.0
Present	0 (0%)	3 (100%)	
Absent	6 (16.2%)	31 (83.8%)	
Mass borders			FET *P* = 1.0
Ill-defined	4 (16.0%)	21 (84.0%)	
Well-defined	2 (13.3%)	13 (86.7%)	
Mass			FET *P* = 1.0
Hypoechoic	5 (15.6%)	27 (84.4%)	
Isoechoic	1 (12.5%)	7 (87.5%)	
Calcification			FET *P* = 1.0
Present	2 (16.7%)	10 (83.3%)	
Absent	4 (14.3%)	24 (85.7%)	
Pathological features			
Site			MC *P* = 0.220
One lobe	6 (21.4%)	22 (78.6%)	
Two lobes	0 (0%)	10 (100%)	
Isthmus	0 (0%)	2 (100%)	
Size (cm)			FET *P* = 1.0
≤ 2	3 (16.7%)	15 (83.3%)	
> 2	3 (13.6%)	19 (86.4%)	
The other lobe			MC *P* = 0.120
Free	2 (10.5%)	17 (89.5%)	
MNG	4 (40.0%)	6 (60.0%)	
Malignancy	0 (0%)	10 (100%)	
Lymphocytic thyroiditis	0 (0%)	1 (100%)	
Capsule			FET *P* = 0.216
Free	5 (21.7%)	18 (78.3%)	
Invaded	1 (5.9%)	16 (94.1%)	
Lymph vascular invasion			FET *P* = 1.0
Present	3 (13.0%)	20 (87.0%)	
Absent	3 (17.6%)	14 (82.4%)	
Multifocality			FET *P* = 0.02*
Unifocal	6 (30.0%)	14 (70.0%)	
Multifocal	0 (0%)	20 (100%)	
Extra thyroid extension			FET *P* = 1.0
Present	1 (14.3%)	6 (85.7%)	
Absent	5 (15.2%)	28 (84.8%)	
Type			MC *P* = 0.019*
Classic	3 (8.6%)	32 (91.4%)	
Cribriform	1 (100%)	0 (0%)	
Follicular	2 (50%)	2 (50%)	
TNM Stage			MC *P* = 0.246
I	6 (21.4%)	22 (78.6%)	
II	0 (0%)	7 (100%)	
III	0 (0%)	5 (100%)	
LN			FET *P* = 0.373
Present	2 (8.7%)	21 (91.3%)	
Absent	4 (23.5%)	13 (76.5%)	
Metastasis			FET *P* = 0.607
Present	6 (16.7%)	30 (83.3%)	
Absent	0 (0%)	4 (100%)	
Recurrence			FET *P* = 1.0
Present	2 (11.8%)	15 (88.2%)	
Absent	4 (17.4%)	19 (82.6%)	

χ^2^: Chi-square test (count and percent are used to display data); FET: Fisher exact test (count and percent are used to display data); t: Separate samples t-test (mean ± SD is whether the data are displayed); Z: Mann-Whitney U test (median and IQR are used to display the data); * *P* ≤ 0.05 indicates a significant difference. *Abbreviations: Long non-coding RNA (lncRNA); prostate cancer-associated transcript 1 (PCAT1); body mass index (BMI) red blood cells (RBCs); white blood cells (WBCs); TNM stands for tumor size; N for nodal metastasis; M for distant metastasis; Hg for hemoglobin; and MNG for multinodular goiter*

Patients were divided into groups with low (≤ 0.80) and high (> 0.80) expression levels of FENDRR. Low expression of FENDRR was found in 80% (32/40) of the PTC specimens. Serum TSH levels (*p* = 0.048), WBC count (*p* ≤ 0.001), lymphocytic percentage (*p* = 0.019), recurrence rate (*p* = 0.05), and BMI (*p* = 0.007) were all substantially correlated with low FENDRR expression. The other features did not significantly correlate with PCAT-1/FENDRR expression (*p* > 0.05) (Table 4).

**Table 4:** Association between FENDRR gene expression and patient characteristics

Parameters	FENDRR lncRNA expression (fold change)		Test of significance
	**Low (≤ 0.8) (n = 32)**	**High (> 0.8) (n = 8)**	
Clinic-demographic data			
Age (Years)			χ^2^ = 0.502 *P* = 0.479
< 55 y	24 (82.8%)	5 (17.2%)	
≥ 55 y	8 (72.7%)	3 (27.3%)	
Sex			χ^2^ = 0.268 *P* = 0.605
Male	9 (75.0%)	3 (25.0%)	
Female	23 (82.1%)	5 (17.9%)	
BMI	29.65 ± 6.01	37.08 ± 3.85	t = 2.87 *P* = 0.007*
Laboratory investigations			
Serum total T3 (*nmol/L*)	1.42 (0.90–2.99)	1.16 (0.46–1.54)	Z = 1.32 *P* = 0.186
Serum total T4(μg/dL)	8.25 (1.97–13.60)	6.11 (3.13–10.40)	Z = 0.754 *P* = 0.451
Serum TSH level (mIU/L)	1.62 (0.0-3.20)	36.71 (0.86–70.30)	Z = 1.98 *P* = 0.048*
WBC count (×10^3^ cells/µL)	8.22 ± 2.42	12.22 ± 3.21	t = 3.91 *P* ≤ 0.001*
RBC count (×10^6^ cells/µL)	4.21 ± 0.86	4.20 ± 0.82	t = 0.031 *P* = 0.975
Hemoglobin (g/dL)	11.05 ± 1.81	11.24 ± 1.94	t = 0.257 *P* = 0.799
Platelet count (×10^3^ cells/µL)	261.56 ± 93.95	252.75 ± 63.61	t = 0.250 *p* = 0.804
Lymphocytic percent (%)	30.39 ± 12.67	17.50 ± 13.53	t = 2.46 *P* = 0.019*
Radiological features			
Retrosternal extension			FET *P* = 1.0
Present	2 (66.7%)	1 (33.3%)	
Absent	30 (81.1%)	7 (18.9%)	
Mass borders			χ^2^ = 2.67 *P* = 0.102
Ill-defined	22 (88.0%)	3 (12.0%)	
Well-defined	10 (66.7%)	5 (33.3%)	
Mass			χ^2^ = 0.156 *P* = 0.693
Hypoechoic	26 (81.2%)	6 (18.8%)	
Isoechoic	6 (75.0%)	2 (25.0%)	
Calcification			χ^2^ = 1.46 *P* = 0.228
Present	21 (75.0%)	7 (25.0%)	
Absent	11 (91.7%)	1 (8.3%)	
Pathological features			
Site			MC *P* = 0.779
One lobe	23 (82.1%)	5 (17.9%)	
Two lobes	7 (70.0%)	3 (30.0%)	
Isthmus	2 (100%)	0 (0%)	
Size (cm)			FET *P* = 0.709
≤ 2	15 (83.3%)	3 (16.7%)	
> 2	17 (77.3%)	5 (22.7%)	
The other lobe			MC *P* = 0.336
Free	14 (73.7%)	5 (26.3%)	
MNG	10 (100%)	0 (0%)	
Malignancy	7 (70.0%)	3 (30.0%)	
Lymphocytic thyroiditis	1 (100%)	0 (0%)	
Capsule			FET *P* = 1.0
Free	18 (78.3%)	5 (21.7%)	
Invaded	14 (82.4%)	3 (17.6%)	
Lymph vascular invasion			FET *P* = 0.250
Present	20 (87.0%)	3 (13.0%)	
Absent	12 (70.6%)	5 (29.4%)	
Multifocality			FET *P* = 0.235
Unifocal	18 (90.0%)	2 (10.0%)	
Multifocal	14 (70.0%)	6 (30.0%)	
Extra thyroid extension			χ^2^ = 0.173 *P* = 0.677
Present	6 (85.7%)	1 (14.3%)	
Absent	26 (78.8%)	7 (21.2%)	
Type			MC *P* = 0.337
Classic	29 (82.9%)	6 (17.1%)	
Cribriform	1 (100%)	0 (0%)	
Follicular	2 (50%)	2 (50%)	
TNM Stage			MC *P* = 0.339
I	21 (75.0%)	7 (25.0%)	
II	7 (100%)	0 (0%)	
III	4 (80.0%)	1 (20.0%)	
LN			FET *P* = 0.107
Present	16 (69.6%)	7 (30.4%)	
Absent	16 (94.1%)	1 (5.9%)	
Metastasis			FET *P* = 0.566
Present	4 (100%)	0 (0%)	
Absent	28 (77.8%)	8 (22.2%)	
Recurrence			FET *P* = 0.05*
Present	11 (64.7%)	6 (35.3%)	
Absent	2 (8.7%)	21 (91.3%)	

χ^2^: Chi-square test (results are shown as count and percent); t: Independent samples t-test (data are presented as mean ± SD); Z: Mann-Whitney U test (data are presented as median and IQR); FET: Fisher exact test (data are provided as count and percent); MC: Monte Carlo test (data are presented as count and percent) * *P* ≤ 0.05 indicates a significant difference. *Abbreviations: Fetal-lethal non-coding developmental regulatory RNA (FENDRR); long non-coding RNA (lncRNA); body mass index (BMI); Hg stands for hemoglobin; MNG for multinodular goiter; TNM for tumor size; N for nodal metastasis; and M for distant metastasis*

### Survival analysis (follow-up and risk of recurrence)

Patients were monitored for up to 60 months to determine their risk of recurrence. The time between the date of surgery and the date of recurrence was referred to as recurrence-free survival (RFS). Patients were excluded based on their final follow-up or date of death if recurrence was not identified. According to overall recurrence-free survival, the 40 PTC patients had an average overall RFS of 44.025 months (95% CI: 36.72–51.32). Using the log-rank test, the Kaplan-Meier survival analysis reveals a significant difference in RFS between patients with low and high expression of FENDRR. RFS was substantially shorter in patients with low FENDRR expression (41.656 months; 95% CI: 32.87–50.43) than in patients with high FENDRR expression (40.643 months; 95% CI: 39.34–59.89) (*p* = 0.024). This suggests that in PTC patients, decreased FENDRR expression is a risk factor for a poor prognosis on its own. Clinicopathological factors associated with shorter RFS shows the presence of calcifications (50.250 months; 95% CI: 31.51–49.77; *p* = 0.046), a tumor size > 2 cm (38.636 months; 95% CI: 27.89–49.37; *p* = 0.023), malignancy in the other lobe (8.000 months; 95% CI: 8.00–8.00; *p* = 0.017), capsular invasion (33.176 months; 95% CI: 20.52–45.82; *p* = 0.009), advanced stage III (10.000 months; 95% CI: 6.90–13.09; *p* ≤ 0.001), and lymphovascular invasion (36.913 months; 95% CI: 26.38–47.44; *p* = 0.034). These factors were identified as independent prognostic factors in the univariate analysis (Table 5).

**Table 5:** Kaplan-Meier analysis for recurrence-free survival

	Recurrence-free survival				
	Mean survival time	Std. Error	95% CI	Log Rank test	*P* - value
Age classes				2.46	0.116
< 55 y	46.586	3.982	38.78–54.39		
> 55 y	37.273	8.138	21.32–53.22		
Sex				1.48	0.224
Male	39.167	7.712	24.05–54.28		
Female	46.107	4.096	38.08–54.13		
Retrosternal extension				0.121	0.728
Present	45.000	12.247	20.99-69.00		
Absent	43.946	3.902	36.29–51.59		
Mass borders				1.715	0.190
Ill-defined	36.840	4.951	27.13–46.54		
Well-defined	56.000	3.445	49.24–62.75		
Mass				0.071	0.789
Hypoechoic	44.562	4.159	36.41–52.71		
Isoechoic	41.875	8.296	25.61–58.13		
Calcification				3.97	0.046*
Present	40.643	4.659	31.51–49.77		
Absent	51.917	5.234	41.65–62.17		
Site				0.066	0.967
One lobe	42.679	4.698	33.47–51.88		
Two lobes	44.600	6.688	31.49–57.71		
Isthmus	60.00	0.000	60.00–60.00		
Size (cm)				5.19	0.023*
< 2	50.611	4.324	42.13–59.08		
> 2	38.636	5.480	27.89–49.37		
Other lobe				10.22	0.017*
Free	52.263	4.599	43.25–61.27		
MNG	41.900	7.429	27.33–56.46		
Lymphocytic thyroiditis	34.100	7.519	19.36–48.83		
Malignancy	8.000	0.000	8.00–8.00		
Isthmus				0.762	0.683
Free	42.846	4.872	33.29–52.39		
MNG	37.375	8.569	20.57–54.17		
Malignancy	58.000	1.155	55.73–60.26		
Capsule				6.82	0.009*
Free	52.043	3.498	45.18–58.90		
Invaded	33.176	6.454	20.52–45.82		
Lymph vascular				4.05	0.034*
Present	36.913	5.372	26.38–47.44		
Absent	53.647	3.751	46.29–60.99		
Multifocality				0.572	0.449
Unifocal	43.700	5.127	33.65–53.74		
Multifocal	44.350	5.369	33.82–54.87		
Extra thyroid				0.051	0.822
Present	38.429	9.461	9.88–56.97		
Absent	45.212	4.008	37.35–53.06		
Stage				19.43	≤ 0.001*
I	46.107	4.278	37.72–54.49		
II	60.000	0.000	60.0–60.0		
III	10.000	1.581	6.90-13.09		
LN				1.57	0.210
Present	42.174	5.117	32.14–52.20		
Absent	46.529	5.267	36.21–56.85		
PCAT1				0.250	0.617
Low expression	47.500	7.887	32.04–62.9		
High expression	43.412	4.149	35.28–51.5		
FENDRR				5.07	0.024*
Low expression	41.656	4.481	32.87–50.43		
High expression	50.250	5.565	39.34–59.89		
Overall recurrence-free survival	44.025	3.725	36.72–51.32	-	*-*

Log Rank (Mantel-Cox) was used. Abbreviations: Confidence interval (CI); multinodular goiter (MNG); prostate cancer-associated transcript 1 (PCAT1); and fetal-lethal non-coding developmental regulatory RNA (FENDRR)

A Multivariate Cox proportional hazard model was applied for factors that achieved significance in univariate analysis. Reduced expression of the FENDRR gene (HR, 3.72; 95% CI: 1.20- 11.49), tumor size more than 2 cm (HR, 3.36; 95% CI: 1.09–10.33), other lobe malignancy (HR, 2.06; 95% CI: 1.19–3.53), and invaded capsule (HR, 3.45; 95% CI: 1.26–9.38) were all significant independent prognostic factors of PTC recurrence (*p* = 0.023, 0.034, 0.009, and 0.015, respectively). (Table 6).

**Table 6:** Multivariate Cox proportional hazards regression analysis

	Cox regression analysis				
	β	SE	HR	95% CI	*P* - value
Calcification	1.372	0.753	3.94	0.90- 17.26	0.069
Size > 2 cm	1.213	0.573	3.36	1.09–10.33	0.034*
Other lobe (Mg)	0.721	0.276	2.06	1.19–3.53	0.009*
Capsule (Invaded)	1.238	0.510	3.45	1.26–9.38	0.015*
Lymph vascular invasion	0.601	0429	1.82	0.788–4.23	0.161
Stage (III)	0.586	0.344	1.79	0.915–3.53	0.089
FENDRR Low expression	1.313	0.576	3.72	1.20- 11.49	0.023*

Standard error (SE), hazard ratio (HR), regression coefficient (β), and 95% confidence interval (CI)

## Discussion

Thyroid cancer is the most common endocrine malignancy globally. Gaining insights into the molecular mechanisms that drive its development and progression is essential for advancing diagnosis, prognosis, and therapeutic approaches. Research on gene expression has shed light on the intricate biological processes involved in thyroid cancer.

Although PTC generally exhibits a favorable overall survival rate with standard treatment, concerns remain regarding the high recurrence rate, which can reach up to 20% during long-term follow-up [15]. Additionally, determining the optimal initial surgical approach and postoperative therapeutic strategies such as whether radioiodine ablation is necessary after total thyroidectomy remains challenging for PTC patients [16].

The ability to rapidly and accurately differentiate benign from malignant thyroid nodules is critical. Given the high prevalence of thyroid nodules in the general population, including potential organ donors, molecular tools such as lncRNA expression profiling could provide valuable support for clinical decision-making. Recent studies emphasize the importance of integrating novel diagnostics into the assessment of thyroid pathology to improve accuracy while adhering to tight clinical timelines [17, 18]. Our findings contribute to this growing field by identifying potential lncRNA markers that may assist in rapid and reliable evaluations.

LncRNAs are now known to be important regulators of gene expression and cellular processes. Instead of producing proteins like protein-coding genes do, lncRNAs alter gene expression through a variety of processes, including transcriptional control, post-transcriptional regulation, and chromatin remodeling [19]. The expression of these biomolecules usually occurs at low levels and has been associated with the progression of several diseases, including TC [20]. Recently, lncRNAs have been described as valuable biomarkers for thyroid cancer patients who may improve diagnosis and prognosis [21].

There is a lack of in-depth information regarding the general role of lncRNAs in thyroid carcinoma since much of the research conducted to date has concentrated almost exclusively on a small group of well-characterized lncRNAs, such as HOTAIR and MALAT1 [20]. In an attempt to fill this information gap, two single lncRNAs, namely, FENDRR and PCAT-1, have been examined for their relative expression levels in PTC patients and a group of controls. Our aim was to investigate their potential role in thyroid cancer pathogenesis and their possible use as diagnostic and prognostic markers in thyroid cancer. To the best of our knowledge, this is the first study to examine the specific roles of FENDRR and PCAT-1 lncRNAs in thyroid carcinoma.

Our results revealed that, in comparison with controls, PTC patients exhibited a lowered count of red blood cells (RBCs), lymphocyte percentages, and hemoglobin levels, while increased serum total T3, white blood cells (WBCs), and platelets. All such discrepancies can most likely be attributed to metabolic, immunologic, and inflammatory derangements in cases of cancer that are not detected in goiter, a non-malignant disease of the thyroid gland. These findings are consistent with Martin et al., whose study revealed that platelet count might be a promising, independent, cost-effective predictor for differentiating PTC from benign thyroid disorders. This easily measurable parameter, with its ease of access and availability, may offer a practical and economical tool for aiding in PTC diagnosis [22].

Our study revealed significantly higher PCAT-1 lncRNA expression in the PTC group than in the control group. PCAT-1 is deregulated in a wide range of human malignancies and is consistently involved in various steps of carcinogenesis such as initiation, proliferation, invasion, and chemoresistance. It has also been closely linked with clinicopathologic factors associated with cancers including tumor size and stage and tumor metastasis, in addition to patient prognosis [23].

Our findings are in agreement with those of a study by Prensner et al. (2011), which investigated PCAT-1 expression in prostate cancer and found that the overexpression of PCAT-1 was associated with prostate cancer progression [11]. This result was also supported by Wen et al. (2016) study who reported that PCAT-1 expression in HCC tissue samples and HCC cell lines was higher compared to adjacent non-cancerous tissue and normal hepatic epithelial cells. Wen et al. (2016) demonstrated that PCAT-1 promotes proliferation, migration, and cell death in HCC, and, by doing so, exerts an oncogenic effect [24]. Several other studies have discussed the association between PCAT-1 and a variety of types of human malignancies, such as hepatocellular carcinoma [25, 26], gastric carcinoma [27, 28], colorectal carcinoma [29], esophageal carcinoma [30], cervical carcinoma [31] and osteosarcoma [32, 33]. These findings suggest that PCAT-1 may play a similar oncogenic role in PTC; hence, its function needs to be studied extensively for its application in therapeutic interventions [24]. Its participation in these vital pathways emphasizes both its potential to promote cancer progression and its significance as a target for therapeutic intervention.

Conversely, our investigation showed that in comparison with controls, PTC patients exhibited reduced expression of the FENDRR gene at a significant level. Consistent with a study conducted by Li et al. (2023), reduced expression of FENDRR is correlated with a poor prognosis in colorectal cancer (CRC). According to their work, FENDRR could have a protective role in CRC development, acting in a tumor-suppressive manner. Thus, in view of its potential, we suggest that FENDRR can serve as a therapeutic target for future studies, with future use in developing new therapeutic approaches for enhancing CRC patient prognosis. Much work is yet to be conducted in its thorough investigation of its mechanism and therapeutic value [34].

Numerous studies have proven that FENDRR is downregulated in many types of cancers, such as lung cancer [35, 36], breast cancer [37], prostate cancer [38], colon cancer [39, 40], hepatocellular carcinoma [41, 42], gastric cancer [43], and cervical cancer [44]. These observations imply that FENDRR could function as a tumor suppressor by inhibiting cancer development.

One of the diagnostic dilemmas of thyroid cancer is the presence of nuclear grooving, an important diagnostic criterion in PTC, in some cases of benign thyroid lesions, causing mis differentiation between the two lesions and, sometimes, thyroid carcinoma misdiagnosis [7]. To evaluate whether lncRNA expression levels can accurately differentiate between PTC and benign tumors, an analysis of an ROC curve was conducted. For both expressions of PCAT1 and FENDRR, analysis of the area under the curve (AUC) determined that the test exhibited high accuracy in differentiation between goiter and PTC, and its use in diagnosing PTC can be supported.

Since as much as 30% of thyroid cancer patients are presented with poor clinical outcomes [4–6], our study also aimed to investigate the prognostic value of the studied lncRNAs in predicting PTC recurrence. The study results revealed that low FENDRR expression is an important, independent poor prognosis marker in PTC, supported by the higher risk of recurrence and shorter recurrence-free survival in subjects with low expression of the gene for FENDRR. Li et al. (2023) reported that gastric carcinoma subjects with low expression of FENDRR have a poor prognosis with high recurrence and shorter overall survival in comparison with subjects with high expression of FENDRR [34]. All these observations validate that FENDRR is a sound prognostic marker in a range of types of malignancies.

Previous studies have indicated that the lncRNA PCAT-1 may regulate the c-Myc signaling axis and impair the DNA damage response, contributing to tumor progression [45]. Similarly, FENDRR has been reported to interact with epigenetic regulators such as Polycomb Repressive Complex 2 (PRC2) and modulate pathways including Wnt/β-catenin and TGF-β signaling [46], which are critical for cell proliferation and differentiation. Although our study did not directly investigate these mechanisms, these insights suggest potential pathways through which PCAT-1 and FENDRR could exert their biological effects in thyroid cancer.

In conclusion, this study is the first to explore the gene expression analysis of lncRNAs PCAT1& FENDRR in thyroid carcinoma. Our findings strongly suggest that both PCAT1& FENDRR may potentially play a role in thyroid carcinogenesis and could be a useful biomarkers in differentiating PTC from benign thyroid lesions. In addition, the FENDRR gene expression may serve as a promising tool for predicting PTC recurrence, particularly, those who express less FENDRR. The functions of both lncRNA and therapeutic potential in the treatment of thyroid cancer require more investigations. Their discovery and further examination expand the list of lncRNAs with potential roles in cancer biology.

### Limitations & recommendations

This impaired expression of lncRNAs PCAT1 and FENDRR is significantly related to many clinicopathological characteristics and patient survival, suggesting that they are potential clinical biomarkers. However, small sized samples make some of our results insignificant. Moreover, the expression and the role of these lncRNAs in body fluids remain poorly understood, and additional large-scale, multicenter studies are necessary to validate these lncRNAs for clinical applications.

This study did not evaluate autoimmune thyroid markers such as anti-TPO antibodies. Future investigations should incorporate assessment of autoimmune status to better understand potential relationships between thyroid autoimmunity and lncRNA expression patterns in PTC.

Also, incorporating lymph node tissue samples in future studies could provide additional insight into the metastatic mechanisms of thyroid carcinoma associated with lncRNA expression patterns.

## Data Availability

No datasets were generated or analysed during the current study.
